# Identification of blood biomarkers of rheumatoid arthritis by transcript profiling of peripheral blood mononuclear cells from the rat collagen-induced arthritis model

**DOI:** 10.1186/ar1883

**Published:** 2006-01-10

**Authors:** Jianyong Shou, Christopher M Bull, Li Li, Hui-Rong Qian, Tao Wei, Shuang Luo, Douglas Perkins, Patricia J Solenberg, Seng-Lai Tan, Xin-Yi Cynthia Chen, Neal W Roehm, Jeffrey A Wolos, Jude E Onyia

**Affiliations:** 1 Integrative Biology, Lilly Research Laboratories, Indianapolis, Indiana, USA; 2 Angiogenesis and Tumor Microenvironment Biology, Lilly Research Laboratories, Indianapolis, Indiana, USA; 3 Statistics, Lilly Research Laboratories, Indianapolis, Indiana, USA; 4 Cancer Inflammation and Cell Survival, Lilly Research Laboratories, Indianapolis, Indiana, USA; 5 Platform/CFARS, Lilly Research Laboratories, Indianapolis, Indiana, USA

## Abstract

Rheumatoid arthritis (RA) is a chronic debilitating autoimmune disease that results in joint destruction and subsequent loss of function. To better understand its pathogenesis and to facilitate the search for novel RA therapeutics, we profiled the rat model of collagen-induced arthritis (CIA) to discover and characterize blood biomarkers for RA. Peripheral blood mononuclear cells (PBMCs) were purified using a Ficoll gradient at various time points after type II collagen immunization for RNA preparation. Total RNA was processed for a microarray analysis using Affymetrix GeneChip technology. Statistical comparison analyses identified differentially expressed genes that distinguished CIA from control rats. Clustering analyses indicated that gene expression patterns correlated with laboratory indices of disease progression. A set of 28 probe sets showed significant differences in expression between blood from arthritic rats and that from controls at the earliest time after induction, and the difference persisted for the entire time course. Gene Ontology comparison of the present study with previous published murine microarray studies showed conserved Biological Processes during disease induction between the local joint and PBMC responses. Genes known to be involved in autoimmune response and arthritis, such as those encoding Galectin-3, Versican, and Socs3, were identified and validated by quantitative TaqMan RT-PCR analysis using independent blood samples. Finally, immunoblot analysis confirmed that Galectin-3 was secreted over time in plasma as well as in supernatant of cultured tissue synoviocytes of the arthritic rats, which is consistent with disease progression. Our data indicate that gene expression in PBMCs from the CIA model can be utilized to identify candidate blood biomarkers for RA.

## Introduction

Rheumatoid arthritis (RA) is a chronic autoimmune disease of unknown etiology that affects 0.5–1% of the population [1]. It is a polyarthritis characterized by inflammation, altered humoral and cellular immune responses, and synovial hyperplasia, leading to destruction and subsequent loss of function of multiple joints [1-4]. Although the exact pathogenesis of RA is not fully understood, the immune and inflammatory systems are intimately linked. Studies on affected joints focusing on cartilage, bone, and synovial tissues have yielded important insights into the mechanisms of disease initiation and progression. Initially, T cell recruitment and recognition of autologous or cross-reacting antigens in the joint produce a variety of mediators, some of which facilitate the development of autoantibodies that are detectable in the serum of RA patients [5]. The ensuing inflammatory responses, induced by tumor necrosis factor (TNF)-α and other proinflammatory cytokines, lead to synovial fibroblast hyperplasia, destruction of the extracellular matrix, and eventual damage to the affected joints [5,6]. Although there have been many studies of cells within the arthritic joint, the responses of the peripheral blood leukocytes are not well understood. An examination of the circulating lymphocytes may provide an important alternative perspective of the processes that underlie RA and complement local characterization of affected joints [7].

Circulating leukocytes provide an important source for biomarker discovery for RA. Emerging high content approaches such as genomics and proteomics have radically changed the ways in which biomarkers are being studied [8-10]. The genomic approaches have been used to elucidate the pathogenesis of inflammatory diseases, including RA, and to identify novel drug targets for RA treatment [3,11-15]. In contrast to target tissue biopsy based approaches, which are often limited by restricted access to target tissues, profiling peripheral blood cells has emerged as an attractive biomarker discovery strategy [10,16-22]. Another added advantage to analyzing peripheral blood cells is the fact that blood is a highly dynamic environment, communicating with practically every tissue in the body, and is thus proposed as a 'sentinel tissue' that reflects disease progression in the body [21,23]. Profiling peripheral blood cells has indeed been used to elucidate autoimmune diseases [7,24].

The rat model of collagen-induced arthritis (CIA) has many similarities to RA [25]. In this model (also demonstrable in mice and monkeys), immunization with type II collagen (CII) – the collagen found in joint cartilage – induces T cell activation, anti-CII autoantibody production, and inflammation and joint destruction similar to that observed in human RA [25,26]. Although there are clearly differences between RA and CIA, changes in peripheral blood gene expression during the development of CIA may suggest potential novel biomarkers for RA. This could be of value both in monitoring the effects of drugs on disease progression and in discovering potential biomarkers, particularly for individuals with early RA. The latter is major problem in RA biomarker identification efforts because human studies are often limited by the late diagnosis relative to the early disease onset. Studying CIA with gradual induction of arthritis could potentially reveal early biomarkers for RA. Moreover, gene expression profiling in animal model holds great promise for our understanding of human pathogenesis. For example, profiling gene expression in a rat model of inflammation using SAGE (serial analysis of gene expression) has provided novel insights into mast cell activation [27].

In the present study, we profiled gene expression in rat peripheral blood mononuclear cells (PBMCs) during the development of CIA. We established the method for blood collection, cell fractionation, RNA isolation, and microarray analysis using the Affymetrix GeneChip technology (Affymetrix, Santa Clara, CA, USA). We identified a large number of genes that were differentially expressed between blood from control and arthritic animals. The gene expression signature in blood appeared to correlate with laboratory indices of disease induction. Using bioinformatics and statistical analyses, we identified a subset of putative biomarkers, which were subsequently validated using TaqMan RT-PCR and immunoblot analyses.

## Materials and methods

### Rat collagen-induced arthritis model, blood collection, and peripheral blood mononuclear cell isolation

The protocol for the *in vivo *studies was approved by the Lilly Institutional Animal Care and Use Committee. Adult (approximately 8 weeks old) female Lewis rats weighing approximately 150 g were obtained from Charles River (Wilmington, MA, USA), housed under standard conditions, and given free access to food and water. Animals were acclimated to the holding room for at least 7 days before initiation of the studies. For the induction of CIA, CII (Elastin Products Company, Owensville, MO, USA) was dissolved in sterilized 0.01 mol/l acetic acid (Sigma-Aldrich, St. Louis, MO, USA) to a final concentration of 2 mg/ml. The mixture was stirred at 4°C overnight until the CII was completely dissolved. CII (2 mg/ml) and incomplete Freund's adjuvant were homogenized at a 1:1 ratio using a PowerGen 125 (Fisher Scientific, Pittsburgh, PA, USA). Each rat was injected intradermally at multiple sites on the back with a total of 0.3 ml of the emulsion (day 0). Seven days later (day 7) this immunization protocol was repeated. Induction and severity of arthritis was determined by change in ankle weight, measured using calipers. Based on previous experience, arthritis (as determined by the first signs of redness or swelling of the ankle joints) is observed approximately 12 days after the first CII immunization. By day 21 the inflammatory response in the ankles has reached its peak, and by day 28 there is significant joint pathology. For these reasons, samples were collected on day 0 (baseline), and on days 10, 21, and 28. Ten rats were collected at each time point. We also included non-immunized animals as negative controls on days 10, 21, and 28. Because of the loss of a few samples due to sample processing or raw chip data quality assurance, the actual number of chips that were statistically analyzed were (respectively) 10, 5, 4, and 5 for control rats on days 0, 10, 21, and 28; and 9, 2, and 8 for arthritic rats on days 10, 21, and 28.

For gene expression analysis, on days 0, 10, 21, and 28, a volume of 3–5 ml blood from individual animals at time of sacrifice was collected by cardiac puncture into heparinized vacutainer tubes (Becton Dickenson, San Jose, CA, USA). Leukocyte counts were determined using a Hemovet 950 (Drew Scientific, Oxford, CT, USA). For PBMC isolation, blood was centrifuged at 1500 *g *for 20 minutes to remove the plasma. The cell pellet was resuspended in Hanks' balanced salt solution (Gibco BRL/Invitrogen, Carlsbad, CA, USA) to the original volume and the cell suspension was carefully layered over the top of 5 ml of Lympholyte-Rat (Cedarlane Labs, Hornby, Ontario, Canada) in a 15 ml Falcon tube. The tubes were centrifuged for 40 minutes at 1500 g and the white cell layer was collected using a Pasteur pipette. PBMCs were rinsed twice with cold Hanks' balanced salt solution and stored in RNA*later *(Ambion Inc., Austin, TX) until RNA isolation.

### RNA isolation and microarray experiments

RiboPure-Blood Kit (Ambion Inc., Austin, TX, USA) was used for isolation of high quality total RNA from PBMCs. After removing RNA*later *by centrifugation, blood cell pellets were lysed in lysis buffer with sodium acetate solution, in accordance with the manufacturer's instruction. RNA was isolated by acid-phenol:chloroform extraction and further purified on a column with glass fiber filter. RNA was then eluted in RNase-free water. Samples were run on a RNA 6000 Nano Gel System (Agilent Technologies Inc., Palo Alto, CA, USA) using Agilent 2100 Bioanalyzer (Agilent) for RNA quality determination. RNA was further purified by using the RNeasy spin column (QIAGEN Inc., Valencia, CA, USA), and then cDNA was generated and labeled for Affymetrix GeneChip according to the standard Affymetrix approach and as previously described [28,29]. Two micrograms of total RNA was used per labeling reaction. cDNA and labeled *in vitro *transcription product were purified using the GeneChip Sample Clean Module (Affymetrix). We obtained an average *in vitro *transcription product yield of about 26.8 ± 9.7 μg/2 μg input RNA, which is sufficient for chip hybridization. Biotin labeled RNA was fragmented and hybridized to rat genome RAE230A chips. Chip processing, image capturing, and raw data analyses were performed using the Affymetrix Microarray Suite MAS5. Probe set signal intensities of each hybridized gene chip were extracted using MAS5 and were normalized using all probe sets to reach the overall 2% trimmed mean of 1,500 for each chip. Chip performance of both control and arthritic samples met standard quality assurance criteria. The chips had an average background of 61.3 ± 8.2, a Raw Q of 2.5 ± 0.4, and percent present call of 46.8 ± 3.3%.

### Statistical analysis to identify differentially expressed genes

The signal intensity data were fitted to an analysis of variance (ANOVA) model to compare the CIA treated samples with control samples at each time point. For a particular probe set, let Y_ijk _be the normalized signal of sample k in treatment j at time I (specifically, i = 1, 2, 3, and 4 for days 0, 10, 21, and 28, respectively; j = 1 and 2 for control and CII injected rats, respectively; and *k *= 1 ... 10 for rats in each treatment group at each time point). The data were fitted to the following statistical model:

Y_ijk _= μ + β_i _+ τ_j _+ β τ_ij _+ ε_ijk_, ε_ijk _~ N(0,σ^2^)

This ANOVA model uses data from all the samples for each probe set to estimate accurately the sample variance to reach robust hypothesis testing. It applies the time effects of sample collection for both CIA and control animals when identifying changes in gene expression after CII injection. This model allows identification of gene expression changes between CIA and control samples at each matched time points, as well as gene expression changes over time in the control samples. The gene expression fold change is the ratio of the average signals of samples in the comparison (for example, treated/control); if the fold change is less than 1, then the ratio is reversed and a '-' added (for example, minus control/treated). Data from each probe set were fitted to the above model independently as is done in other studies [30,31].

To control the false positive rate of testing the expression change of thousands of genes simultaneously, false discovery rate (fdrate or FDR) was estimated using an algorithm derived by Benjamini and Hochberg [32]. FDR estimates the false positive rate of a 'significant' gene list. Suppose that *P*_i _(i = 1, 2 ... m) are the *P *values resulting from testing m expression changes. Sort *P*_i _from the smallest to the largest, and let *P*_(i) _be the i^th ^sorted *P *value and i its rank. Then, the FDR for each sorted *P *value was calculated by timing the *P *value with m/i, and monotonizing all of the FDRs from the largest to the smallest:



### Bioinformatics analyses

#### Clustered correlation analysis

Cluster correlation analysis was performed with an R script written in-house, in accordance with the method proposed by Weinstein and coworkers [33].

#### Ortholog mapping and Gene Ontology analyses

Genbank accessions or gene identifications were retrieved from published papers or online supplementary materials, and their rat orthologs were obtained by querying NCBI HomoloGene database [34]. The Gene Ontology (GO) analysis was carried out by using GoMiner, developed by Weinstein and colleagues [35]. Briefly, retrieved gene symbols were input into GoMiner, which maps them onto the GO tree, in particular the ontology Biological Process, using organism-specific information provided by NCBI GoMiner server. Percentages of differentially expressed genes were calculated for 10 selected entries within the ontology Biological Process at the third or fourth GO level.

### Quantitative real-time RT-PCR validation

RNA from an independent CIA life phase study was used to validate microarray data. Before cDNA synthesis, RNA samples were DNase treated to remove genomic DNA contamination by using Ambion's DNA-free Kit (Ambion Inc., Austin, TX, USA), in accordance with the manufacturer's instructions. cDNA was prepared from total RNA using Superscript III (InVitrogen, Carlsbad, CA, USA) with random primers as described by the manufacturer. Real-time PCR was performed on an ABI 7900HT from Applied Biosystems (ABI, Foster City, CA, USA) with gene expression assays or with primers and probes from Biosource International (Camarillo, CA). Primers and probes were designed using Primer Express (ABI). Briefly, cDNA templates for real-time PCR were prepared by diluting 1:100 with 10 mmol/l Tris (pH 7.5). The 20 μl TaqMan reaction consisted of 1 × Universal Master Mix (ABI), 1 × Gene Expression Assay (ABI), and 4 μl diluted cDNA. TaqMan reactions for genes that were assayed with primers and probes consisted of 1 × Universal Master Mix (ABI), 0.8 μmol/l forward and reverse primers, 0.2 μmol/l probe, and 4 μl diluted cDNA in a final volume of 20 μl.

Five replicates of each RT-PCR reaction were assembled in 384-well plates, on a Tecan Genesis 150 (Maennedorf, Switzerland) liquid handling robot. Each plate included no RT controls for each sample and no template control. Raw data were analyzed using a macro created in Microsoft Excel. Briefly, the high and low values from each of the five replicates were discarded and the remaining three values averaged. The average values were normalized to 18s rRNA relative expression values. Data analysis was conducted in JMP 5.1.1 (SAS Institute, Cary, NC, USA). Best Box-Cox transformation was used in order to fit the model. For comparing the means of groups with the control group, the data for different time points were tested through Dunnet's test. Conventional alpha (a = 0.05) is regarded as significant.

Gene expression assays (ABI) were included for the following genes: Galectin-3 (Lgals3, Rn_00582910_m1) and Cish3 (Rn00585674_s1). Primers and probes for Versican (Cspg2) and IL-6 were purchased from Biosource International. Sequences for the Cspg2 primers were as follows: forward, 5'-CGCCTAAGACACTACGTATGCTTGT-3'; reverse, 5'-TTGGTCCTATGTTGACTGTTTCTCA-3'; and probe, 5'-AGCATAGTCATTCCCTCTAAGCCAAAGAAGGTTC-3', labeled with 6-FAM and BHQ-1. IL-6 primers were as follows: forward, 5'-CATAGTCGTGCCTGTGTGCTTAG-3'; reverse, 5'-AGGTCTCGTTTATTAAAGCAGAACAAG-3'; and probe, 5' TTTCCTCCTGACAACGCTGCTGGG-3', labeled with 6-FAM and BHQ-1.

### Synovial tissue culture and Western blot analysis for Galectin-3

Synovial tissue from the arthritic rats at different times after CII immunization were dissected and collected in the collecting medium (Dulbecco's modified Eagle's medium + 0.5% penicillin/streptomycin and antimycotics; Gibco-BRL/Invitrogen). The tissue was washed two times with the collecting medium and one time with the culture medium (Dulbecco's modified Eagle's medium + 10% heat inactivated fetal calf serum and 1% penicillin/streptomycin; Gibco-BRL/Invitrogen). The synovial tissue was then placed immediately into a 24-well tissue culture plate (two pieces of synovium in 1 ml medium per well) with culture medium, and cultured in 5% carbon dioxide at 37°C for 48 hours. The culture plate was centrifuged at 1500 rpm for 10 minutes at 4°C. The supernatant was collected and stored under -80°C until the assay.

Plasma or supernatant from cultured tissue synoviocytes of the CIA rats was subjected to Western blotting using NuPage 4–12% Bis-Tris gels, MOPS running buffer, transfer buffer, and 0.2 μm PVDF membrane (Invitrogen), in accordance with the manufacturer's protocol. Monoclonal antibody to Galectin-3 antibody (A3A12; cat. no. 804-284-C100) was purchased from Alexis Biochemicals (San Diego, CA, USA). Recombinant mouse Galectin-3 protein (cat. no. 1197-GA; R&D Systems, Minneapolis, MN, USA) was used as positive control. The blots were developed using SuperSignal West Femto Maximum Sensitivity Substrate from Pierce (Rockford, IL, USA).

## Results

### Gene expression profiling in peripheral blood mononuclear cells in the collagen-induced arthritis model

**Figure 1:**
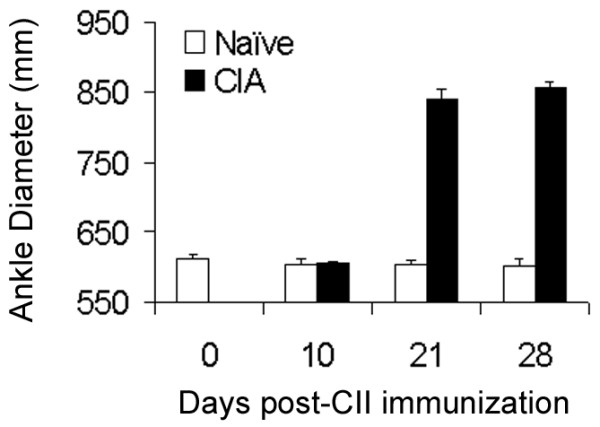
Inflammatory response in the ankles of rats during the development of CIA. Ankle diameters were measured in naïve (*n *= 5) and CII immunized (*n *= 10) rats on the indicated days, before blood collection and sacrifice of the animals. Each time point represents a different set of animals. CIA, collagen-induced arthritis; CII, collagen type II.

To identify putative biomarkers for arthritis, we surveyed global gene expression profiles of PBMCs in a rat CIA model using DNA microarray technology. We assayed PBMCs from animals sacrificed at days 10, 21, and 28 after the first CII immunization and day 0 naïve rats. These time points were chosen based on the pathological development of disease in this model. Changes in ankle diameter (a measure of inflammation) in the different groups are presented in Figure 1.

We applied statistical analyses to examine the difference in gene expression between the control and arthritic rat blood samples. We considered FDR 0.05 to be significant (for example, of the selected 'significant' probe set list, 95% are expected to be real positives). We further trimmed down the probe set list by applying empirical criteria of fold change at least 1.4 (increase or decrease) and mean signal difference at least 250, in order to reduce errors pertained to low-level expression at close to noise level. In addition, in this experiment we had time-matched naïve control samples at each time point, so we could assess the gene expression changes over time in the control animals, or basal expression variation.

**Figure 2:**
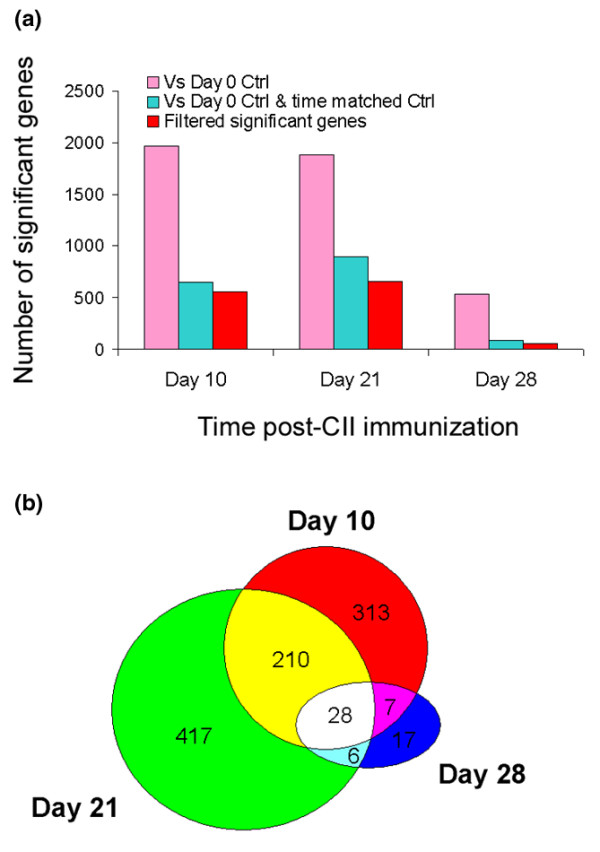
Identification of differentially expressed genes between the rats with CIA and the control rats. **(a) **Number of significantly changed probe sets over time. Statistical pair-wise comparisons and empirical filtering were applied to identify differentially expressed genes (FDR <0.05, fold change >1.4, signal difference >250), as described in the Materials and methods and Results sections. Pink bars represent the number of probe sets that are significantly different from the day 0 control at the indicated time points. Blue bars represent the number of probe sets that are significantly different from the day 0 control as well as the time-matched control at the indicated time points. Red bars represent the number of probe sets that are significantly different from the day 0 control as well as the time-matched control at indicated time points, with the probe sets that fluctuated in control animals excluded. **(b) **Venn diagram of the differentially expressed genes. Probe sets identified as significantly changed genes at each time point were examined for overlapping over time. There are a total of 28 probe sets that significantly changed at all three time points. Note that there is a considerable amount of overlapping between day 10 and day 21; half of the genes identified at day 28 are also included in the day 10 and day 21 gene lists. CII, collagen type II; FDR, false discovery rate.

The control animals at each time point were compared with day 0 control animals. We observed a considerable amount of basal gene expression change, which could be attributable to biologic fluctuation or technical variation. Because we were interested in biomarkers, we focused our analysis on genes with large expression changes after CIA induction but that were relatively stable in the control animals. Thus, we excluded genes that had a large basal expression fluctuation. After excluding the 'fluctuating' probe sets from our significant gene lists, we identified a total of 998 nonredundant probe sets, including 714 known genes that changed significantly at least at one time point. The number of significantly changed probe sets was plotted as a function of time after CII immunization in Fig. 2a. The probe sets and associated annotations are summarized in Additional file 1 for each of the three time points. Venn logic analysis of the 998 probe sets showing the distribution of these genes with respect to time is shown in Figure 2b. We observed a notable amount of overlapping probe sets between day 10 and day 21, but substantially fewer genes were identified for day 28 samples. Nevertheless, almost half (28 out of 58 probe sets) of the day 28 probe sets overlapped with day 10 and day 21. As an initial effort, we focused on genes whose expression changed significantly at all three time points – a list of 28 probe sets that might have a wider time window for assay development. Because of probe set redundancy for Versican/Cspg2, the 28 probe sets actually represented 20 unique known genes and six expressed sequence tags. These 28 probe sets are summarized in Table 1.

### Correlation of gene expression pattern with laboratory indices for disease progression

**Figure 3:**
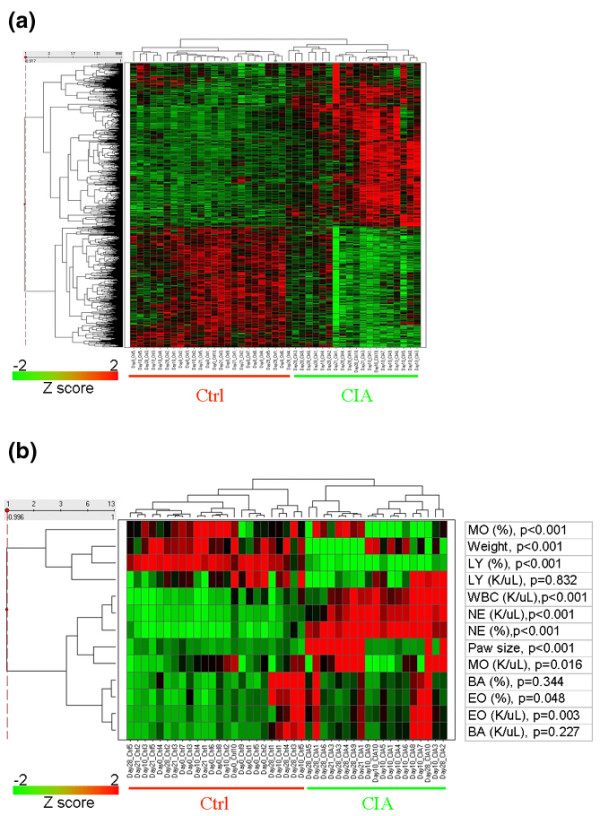
Clustering analyses using gene expression in PBMCs and the laboratory indices of disease progression. **(a) **Hierarchical clustering analysis using 998 nonredundant significant probe sets. The 998 nonredundant significant genes were normalized using Z-score calculation. Genes were clustered in Spotfire DecisionSite (Spotfire, Somerville, MA, USA). The correlation coefficient was used as distance metric and complete linkage was used as the clustering algorithm. **(b) **Hierarchical clustering of laboratory indices of disease progression. The laboratory indices for disease progression were used to cluster the samples. The measurements were normalized using the Z score across different animals and clustered in Spotfire DecisionSite, using the same algorithm as that for gene expression clustering, with correlation coefficient being used as distance metric and complete linkage as the clustering algorithm. The measurements are as follows: animal gross weight (weight), paw size (paw size), total white cell count (WBC), total lymphocyte count (LY), percentage lymphocyte of total WBCs (LY%), total monocyte count (MO), percentage monocyte count (MO%), total neutrophil count (NE), percentage neutrophil count (NE%), total eosinophil count (EO), percentage eosinophil count (EO%), total basophil count (BA), and percentage basophil count (BA%). Statistical tests were performed and the *P *value was attached for each measurement. Note that the phenotypic measurements separated the sample in a similar manner to the gene expression profiles. CIA, collagen-induced arthritis

We next explored the hypothesis that differences in gene expression between the arthritic and the control rat peripheral blood reflect pathological progression in the CIA model. Shown in Figure 3a is a hierarchical clustering analysis using the nonredundant 998 differentially expressed genes (DEGs) identified from the ANOVA analysis. Expression of these 998 probe sets in the arthritic rats was clearly distinct from that in control rats. We next clustered the samples using the normalized laboratory indices including blood cell counts and paw size measurements. The animals were grouped in a manner similar to gene expression clustering (Figure 3b). The total white blood cells, percentage of lymphocytes, and percentage of and total neutrophil counts in arthritic animals were different from those in controls over time. We then performed statistical analysis by fitting the laboratory indices to a similar ANOVA model used for gene expression analysis over the three time points (days 10, 21, and 28). The test showed that the difference between CIA and control animals over the three time points were significant for most of these laboratory measurements. The *P *value for each measurement is shown in Figure 3b.

**Figure 4:**
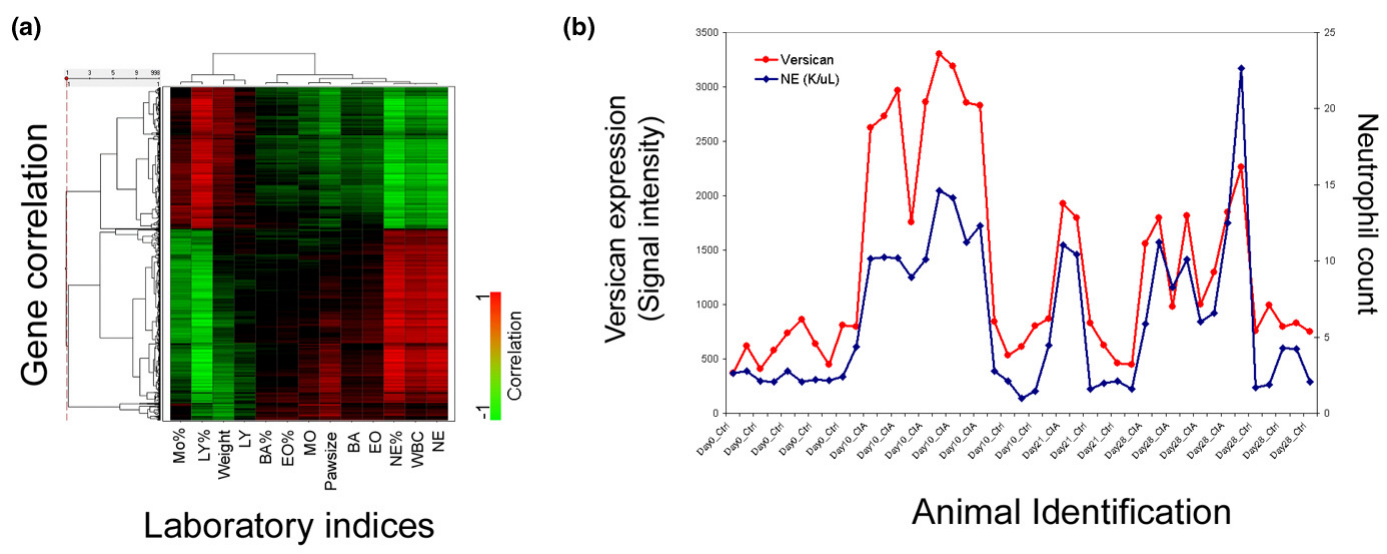
Correlation between gene expression profiles and laboratory indices of disease progression. **(a) **Clustered correlation analysis. Gene expression data were correlated with phenotypic measurements using clustered correlation analysis 33. The correlation coefficient values of each probe set to laboratory measurements were presented in a heat map visualization generated in Spotfire DecisionSite. The measurements are as follows: animal gross weight (weight), paw size (paw size), total white cell count (WBC), total lymphocyte count (LY), percentage lymphocyte of total WBCs (LY%), total monocyte count (MO), percentage monocyte count (MO%), total neutrophil count (NE), percentage neutrophil count (NE%), total eosinophil count (EO), percentage eosinophil count (EO%), total basophil count (BA), and percentage basophil count (BA%). **(b) **Correlation of Versican expression with neutrophil count. The expression level (signal intensity) of Versican from the Affymetrix microarray experiment were plotted, together the neutrophil count (K/μl) for each animal that was used in our microarray study. CIA, collagen-induced arthritis.

In an attempt to explore the possible correlation between gene expression pattern and laboratory indices of disease progression, we integrated the gene expression data with the laboratory indices using clustered correlation analysis [33]. The results are shown in Figure 4a. Details regarding the correlation between each of the 998 DEGs and laboratory indices are summarized in Additional file 2. Remarkably, the 28 probe sets we identified using ANOVA test and Venn logic analysis were among the genes that best correlated with laboratory indices. The gene that exhibited the strongest correlation with total white cell, and total and percentage neutrophil counts was Versican, whereas the gene that negatively correlated with percentage lymphocyte count the best was GIIg15b. Both genes are among the 28 probe sets identified (Table 1). Concordant change between Versican and neutrophil count is shown in Figure 4b as a representative example of the agreement between gene expression and laboratory measurements. Taken together, these data suggest that the gene expression pattern overall correlates with laboratory indices of disease progression.

#### Comparison of the present study with published microarray studies in murine rheumatoid arthritis models

**Figure 5:**
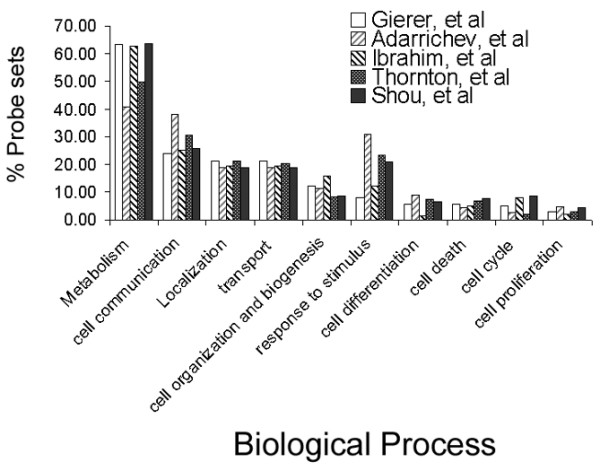
Biologic processes revealed by the present study and previously published murine studies. Genes identified by previous published studies were retrieved from the papers or from online supporting materials [11, 13-15]. Their rat orthologs were obtained by querying NCBI HomoloGene database. The retrieved gene symbols were mapped onto the Gene Ontology (GO) tree, in particular Biological Process, using GoMiner. Percentages of differentially expressed genes were calculated for the selected 10 biological processes at the third or the fourth GO levels and plotted. Note the overall similarity in Biological Process represented by the five independent studies.

We compared our results with the findings of four previous studies conducted in murine autoimmune arthritis models [11,13-15] in order to appreciate better the gene expression in PBMCs in the rat CIA model. We retrieved the reported DEGs from these published studies. Comparisons were made at two levels. First, we compared differentially expressed rat and mouse ortholog genes, which originated from a common ancestor gene and are assumed to play similar biological functions in two distinct species [34]. Of 714 DEGs identified from our study, 70 genes were also identified by at least one other study. Nine of them were identified by at least three studies, including Scos3/Cish3, S100a8, Ptpns1, Lst1, Ctsk, Cd14, Csrp3, App, and Bzrp. Although ortholog gene comparison is relatively easy to interpret, it may not be desirable because of the fact that the different studies were conducted in different conditions, for example using different chip platforms. Thus, we compared our study with the other four studies in terms of the Biological Processes (GO ontology) in which the identified DEGs were involved. Each list of DEGs identified by the different studies was mapped onto the Biological Process GO tree using GoMiner [35]. Percentages of DEGs at each GO category at the third and fourth levels were calculated. Figure 5 shows the percentages of the top 10 Biological Processes in the five studies. Although gene–gene comparison shows relatively little overlap, comparison at higher Biological Processes revealed much greater consistency. For example, the most important Biological Processes include metabolism, cell communication, localization, and transport. Heterogeneous response was only observed in the category of response to stimulus.

#### Functional relevance and validation of putative biomarker candidates

Regulated cytokine expression was reported to be associated with local joints during the development of RA [5]. We surveyed our data for cytokine expression. The expression of cytokine-related probe sets defined by GO are summarized in Additional file 3. Our data indicated that a few cytokines were differentially regulated between arthritic rats and the controls. For example, expression of IL-1β and its type II receptor were significantly upregulated at days 10 and 21, but not at day 28. Our data revealed the involvement of interferon-γ, TNF-α, and transforming growth factor-β signaling pathways during arthritis development in the CIA model, which is consistent with previous studies.

**Figure 6:**
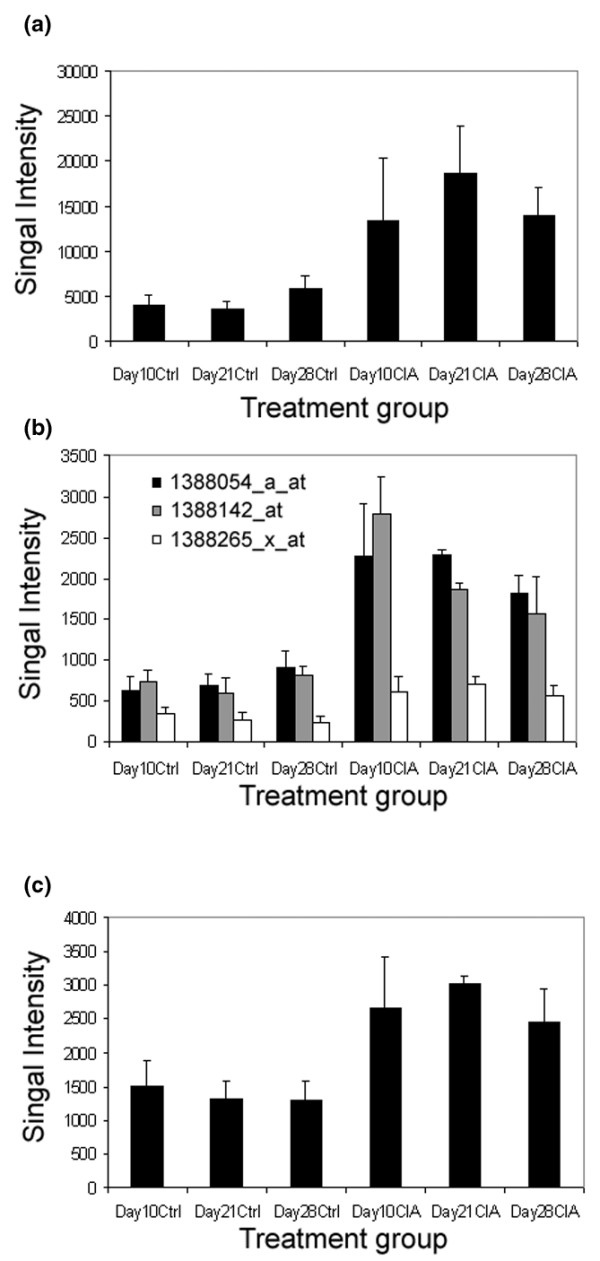
Expression of three selected biomarker candidates of interest. **(a) **Galectin-3, **(b) **Veriscan/Cspg2, and **(c) **Socs3 were selected as putative biomarker candidates of interest. The signal intensity data for these three genes were plotted over time. There are three probe sets for Versican that are significantly different between the arthritic and control samples. Data are expressed as Mean ± standard deviation. Note that expression of these probe sets are low in the control samples, and are upregulated in the arthritic samples at all time points examined. CIA, collagen-induced arthritis.

**Figure 7:**
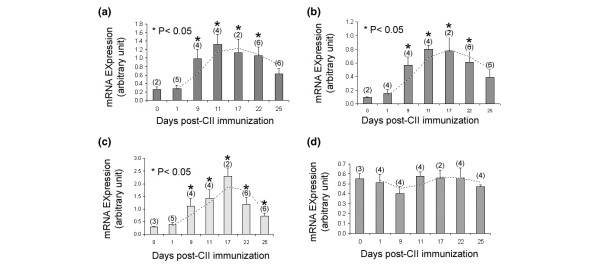
TaqMan validation of the expression of the selected biomarker candidates. TaqMan RT-PCR was performed using primer and probe sets specific to **(a) **Galectin-3, **(b) **Veriscan/Cspg2, and **(c) **Socs3. **(d) **IL-6, an acute responding gene that has not been selected from the microarray analysis, was also assayed as a control. The RNA samples are independent from the ones used for microarray analysis, and more time points were used in the PCR analysis. Data are expressed as mean ± standard error. The number of the samples assayed for each group is marked in the parenthesis above the histogram. * *P *< 0.05, by Dunnet's test. CII, collagen type II.

We focused our initial experimental characterization and validation on three genes: Galectin-3, Versican, and Socs3. They were previously implicated in RA and other immune and inflammatory disorders [24,36-38]. As shown in Figure 6, all three genes were expressed to significantly greater extents in the arthritic animals than in the controls at all three time points, correlating with inflammation and immune responses. To validate our microarray findings, we performed real-time RT-PCR on the three identified candidate biomarker genes using a separate animal cohort with more defined time points to increase validity. The results are shown in Figure 7. The numbers of samples assayed for a given gene at each time point are marked on the histogram. The expression of Galectin-3, Socs3, and Versican over time in the CIA model, as revealed by RT-PCR, agreed well with the microarray data. In contrast IL-6, which is an acute response cytokine [5] and was not identified as a significantly changed gene in our microarray study, did not exhibit significant difference in expression over time by the RT-PCR analysis.

### Immunoblot analysis of Galectin-3 expression in collagen-induced arthritis rat cultured synoviocytes and plasma

**Figure 8:**
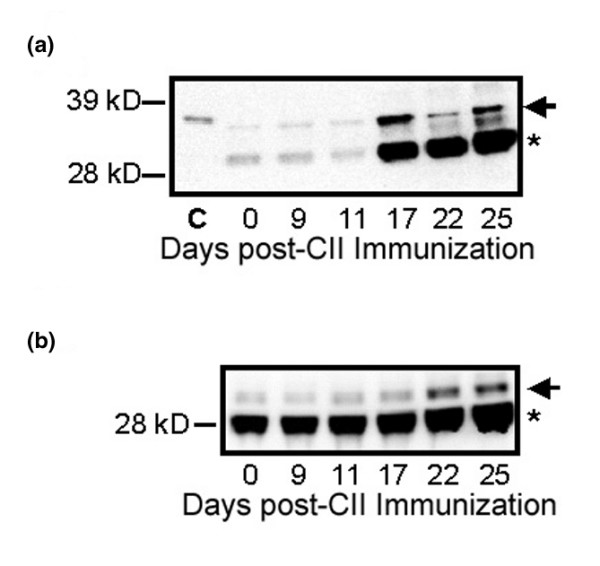
Immunoblot analysis of Galectin-3 in supernatant from cultured synoviocytes or plasma from arthritic rats. Western blot analysis was performed using an anti-Galectin-3 antibody on **(a) **supernatant from cultured tissue synoviocytes or **(b) **plasma samples collected at the indicated time points from arthritic rats. Two nanograms of recombinant mouse Galectin-3 (R&D; cat. no. 1197-GA) was loaded as the positive control (lane c). Arrow denotes rat Galectin-3; asterisk denotes non-specific protein band.

We examined whether the difference in gene expression observed at the mRNA level in PBMCs could be extended to the protein level. We performed Western blot analysis on Galectin-3 using cultured tissue synoviocytes or plasma from the CIA animal cohort that was used for PCR validation. Because Galectin-3 is a secreted protein [36], we first attempted to detect it in the supernatant of cultured tissue synoviocytes. A recombinant mouse Galectin-3 was used as a positive control for the anti-Galectin-3 antibody used in our study. Although the predicted molecular weight of mouse Galectin-3 is 27.3 kDa, the recombinant protein appeared to have a greater molecular mass on the Western blot (Figure 8a). Importantly, a corresponding band was detected in the cell supernatant samples collected at days 17, 22 and 25, but not at the earlier time points. A similar protein expression profile for Galectin-3 was detected in plasma (Figure 8b), further supporting our RNA expression results and the feasibility of developing Galectin-3 as a blood biomarker-based standard protein assay for preclinical and clinical studies.

## Discussion

Biomarkers for RA are much needed if we are to understand and measure disease progression, and to facilitate the development of novel treatments for RA. In the present study we described a noninvasive strategy to discover RA biomarkers by transcript profiling of peripheral circulating lymphocytes. As an initial proof-of-concept, we demonstrated the feasibility of such technology by successful profiling PBMCs in a rat CIA model. We characterized differential gene expression between the normal and arthritic animals, and demonstrated that the gene expression in PBMCs could serve as surrogates that are indicative of disease progression.

We used the combination of statistical ANOVA analysis with clustered correlation and biologic relevance analysis to select a workable number of genes as potential biomarker candidates and to assess the specificity of these marker candidates. We were able to confirm elevated Galectin-3 protein expression in the CIA plasma and cultured synovial tissue [36]. Interestingly, Galectin-3 and its binding protein, but not Galectin-1, were reported to be elevated in RA but not in osteoarthritis [36]. In our study, Galectin-1 was not shown to be elevated in arthritic rat blood either. Thus, blood expression of Galectin-3 is likely to be specific to RA. Socs3 might also be specific to RA [38]. In contrast, Versican/CSPG2 is implicated in osteoarthritic cartilage [37]. Although it was also reported to be over-expressed in PBMCs from RA patients [7,24,39], we speculate that Versican might be involved more in the inflammation responses linked to bone erosion.

The genes we identified also exhibited strong correlation with phenotypic measurements, as demonstrated by the clustered correlation analysis. Versican is the gene exhibiting the strongest correlation with the characteristic measurements, particularly neutrophil count, in the CIA model. Moreover, members of the Galectin family and its binding proteins, Socs3, and Versican are all found to present in human blood (Shou and coworkers, unpublished data). In the future, it will be of great interest to extend these findings to clinical human blood and explore the possibility that these markers could be used to aid preclinical and clinical studies.

The differences between arthritic and control rat blood could result from induction or suppression of gene expression, or could be due to cell type specific gene expression in cell populations recruited to the blood during the development of disease [40] – two alternatives that are very challenging to distinguish. Our cell counting data indicate that total white cell and neutrophil counts, among other parameters, are significantly different between arthritic and control rat blood. Hence, differences in composition or activation state between different types of lymphocytes should contribute to and reflect the differential gene expression that we observed. Our analysis of the correlation between gene expression and laboratory indices might potentially reveal some insights regarding cell type specific gene expression. In the future, it will be of interest to explore further differential cell recruitment and its contribution to gene expression and RA pathogenesis. Additional cell fractionation and small quantity RNA labeling technologies [41,42] will need to be developed to address this issue. Another future direction in evaluating our candidate markers is to monitor the expression of these genes when effective experimental drugs are administrated to CIA rats. It will be important to establish the association between drug effects on inflammation or bone erosion and the expression of the marker genes; this may improve our understanding of drug pharmacokinetics/pharmacodynamics, and facilitate assessment of new compounds for RA treatment, ultimately in a clinical setting.

Major advantages in using the peripheral blood cells instead of local joint tissue to seek biomarkers include the noninvasive nature for the former approach and associated ease preclinical and clinical development [10,20]. Moreover, blood is a highly dynamic system, in which blood cells have a rapid natural turnover (blood cell turnover is estimated at 1 trillion cells/day) [21]. Because the leukocytes interact and communicate with practically every tissue, they bear rich information regarding inflammation and immune responses [23]. Thus, blood – increasingly being recognized as a sentinel tissue – is uniquely suited to study of systematic responses during disease progression. For example, expression in blood of tissue-specific cardiac genes was reported to permit distinction between patients with coronary artery disease and normal control individuals [23]. This strategy has also been successfully applied to the study of cancer biology [17,19], autoimmune disease [7,13,24], cardiovascular disease [43], kidney disease [18], post-traumatic stress disorder [44], and psychiatric disorders [22]. Gene expression profiling in peripheral blood therefore holds great promise for clinical development [10].

In the present study, we demonstrated that gene expression in PBMCs from rats with CIA could distinguish arthritic samples from normal control samples, and that gene expression in PBMCs can indeed serve as a potential candidate biomarker of disease progression. Interestingly, some genes that we identified in PBMCs have also been reported to exhibit altered expression in local joints, suggesting conservation between PBMCs and the local joint tissue in terms of their responsiveness to collagen-induced immunity. The contribution of the genes expressed in PBMCs *per se *to disease progression in CIA and the relevance of these genes to RA is not clear and warrants future investigation. Nevertheless, the present study provides additional evidence supporting the 'sentinel' hypothesis.

A number of genomics studies were previously performed to study RA pathogenesis in murine models [11,13-15,45] or human patients [3,46], with a major focus on local joint tissues. We compared our PBMC profiling findings with those of four published local joint profiling studies using murine models of RA. However, we only identified a limited number of individual genes exhibiting consensus. The observed discrepancy may have multiple causes. First, gene expression in arthritic animal blood is expected to differ substantially from local arthritic joint responses. Second, the difference in technical platforms (for example, spotted array versus the Affymetrix GeneChip, different array versions, and differences in sample preparation and analysis methods) used in these studies may contribute significantly to the difference in DEGs identified. Third, there is only a small portion of the annotated probe sets for which rat orthologs have been identified. Finally, the inherent difference between the murine and rat models of RA may also contribute to the difference in gene expression.

We were able to confirm some known RA related genes in the present study, such as Stat3, Bst1 (bone marrow stromal cell antigen 1), Ptgs2 (prostaglandin G/H synthase 2), S100a9 (S100 calcium binding protein A9) and Ets1 (Ets avian erythroblastosis virus E2 oncogene homolog 1), in addition to chondroitin sulfate proteoglycan 2, Galectin3 and Socs3 (suppressor of cytokine signaling 3). However, we failed to identify some other previously reported RA related genes such as CD36, CD44, STAT5b (signal transducer activator transcription 5b), IL-1Ra follistatin-like genes, IL-13 receptor α, and CCL27 (CC chemokine ligand 27), among others. Interestingly, we identified a IL-1 decoy receptor that antagonizes IL-1 signaling similarly to IL-1Ra, which is known to be involved in RA, suggesting that the consensus could be reached at the gene function level as opposed to the individual gene level. We thus compared our data at a higher level by examining the GO-defined Biological Process represented by the DEGs. We observed a significant degree of agreement between our study and the four previously published ones (Figure 5). The consensus suggested conservation of Biological Processes involved in arthritis development between the local joints and PBMCs, as well as between murine and rat RA models.

The CIA model is a highly dynamic model, with time dependent disease progression. Survey of DEGs identified at various time points can help to improve our understanding of disease development and facilitate biomarker identification. Genes identified at early time points would presumably be informative with respect to early signaling cascades during disease onset. For example, Tnfrsf1b was found to be up regulated in day 10 arthritic rat PBMCs, but not at later time points. Tnfrsf1b encodes a protein with strong similarity to TNF receptor 1b, which induces T cell proliferation and apoptosis. Our data support the involvement of TNF signaling events in the early autoimmune response. Swollen joints are among the important characteristics of arthritis [47-49]. However, paw size measurement only revealed moderate correlation with gene expression in PBMCs (Figure 4). The findings regarding gene expression and correlation with laboratory indices indicate that differences in gene expression in PBMCs between the arthritic rats and control rats, even before the joint swelling, are evident, and thus might be indicative of the early onset of disease. The details of differentially expressed probe sets at different time points are described in Additional file 1. Further characterization of the genes novel to arthritis will advance our understanding of and facilitate identification of novel biomarkers for RA.

## Conclusion

We established a noninvasive strategy to identify biomarkers by gene expression profiling in PBMCs in an experimental model of RA. We characterized the differential gene expression between the normal and arthritic animals, and demonstrated that the gene expression in peripheral blood correlated with laboratory indices of disease progression. Potential biomarker candidates were further validated in independent samples using real-time RT-PCR analysis. Finally, Galectin-3 protein was detected by immunoblot analysis in plasma from CIA rats as well as in supernatants from cultured arthritic rat synovial tissue. Further characterization of the genes novel to arthritis will advance our understanding of and facilitate the identification of novel biomarkers for RA.

## Abbreviations

ANOVA = analysis of variance; CIA = collagen-induced arthritis; CII = type II collagen; DEG = differentially expressed gene; FDR (fdrate) = false discovery rate; GO = Gene Ontology; IL = interleukin; PBMC = peripheral blood mononuclear cell; RA = rheumatoid arthritis; RT-PCR = reverse transcriptase polymerase chain reaction; TNF = tumor necrosis factor.

## Competing interests

The authors declare that they have no competing interests.

## Authors' contributions

JS, HRQ, SLT, NWR, JAW and JEO participated in study design. CMB and LL carried out the life phase animal experiments and blood collection. JS performed the microarray study and drafted the manuscript. HRQ performed the statistical analysis. TW performed the bioinformatics analysis. SL, DP, PJS, and LL were involved in PCR validation and analysis. XYCC and SLT contributed to the Western blot analysis. JS, HRQ, TW, SLT, JAW, and JEO contributed to data interpretation and participated in writing the manuscript. All authors read and approved the final text before submission of the manuscript.

## Supplementary Material

Additional File 1 An Excel file containing a list of probe sets that are differentially expressed by CIA and control rat PBMCs. Click here for file

Additional File 2 An Excel file showing correlations between the 998 differentially expressed probe sets and laboratory indices for disease progression. Click here for file

Additional File 3 An Excel file showing differentially expressed cytokine related probe sets between CIA and control rats. Click here for file

## Figures and Tables

**Table 1:** Genes that changed significantly in all the arthritic rat blood samples

Probe set	Fold change (CIA/control)			Gene description
		
	Day 10	Day 21	Day 28	
1367612_at	4.94	4.31	2.10	Mgst1: microsomal glutathione S-transferase 1
1367816_at	1.89	2.58	1.51	GIIg15b: protein similar to 2300002F06Rik
1367900_at	4.93	4.56	2.93	Gyg: glycogenin (glycogenin glucosyltransferase)
1367904_at	--1.84	--1.76	--1.49	Resp18: regulated endocrine-specific protein 18
1369584_at	1.76	2.32	1.91	Socs3 (Cish3): suppressor of cytokine signaling 3
1369956_at	2.81	2.82	1.98	Ifngr: similar to interferon gamma receptor
1370119_at	3.10	2.73	1.86	Lst1: member of the LST-1 protein family
1370249_at	3.01	3.99	1.92	Bzrp: peripheral-type benzodiazepine receptor
1371916_at	2.64	3.29	1.60	Sepr: selenoprotein R
1372150_at	--2.24	--2.39	--1.72	Usp10: human ubiquitin specific protease 10 like
1372248_at	--1.88	--3.11	--1.76	SESN1: p53 regulated PA26 nuclear protein
1372691_at	4.46	6.19	2.32	Upp1: uridine phosphorylase 1
1373656_at	2.83	4.03	1.74	--
1374375_at	3.45	5.60	2.21	2610034M16Rik
1377092_at	3.61	2.38	3.71	
1377110_at	--1.49	--2.60	--1.48	Plxdc1: plexin repeat containing family member
1386052_at	--1.80	--2.70	--1.58	
1386879_at	3.35	5.20	2.36	Lgals3: Galectin-3
1386908_at	2.66	2.32	1.61	Glrx1: Glutaredoxin
1387568_at	3.68	4.65	1.82	Pirb: paired immunoglobulin-like receptor-B
1387599_a_at	2.73	4.12	1.76	Nqo1: NADH:NADPH diaphorase
1388054_a_at	3.64	3.31	1.98	Cspg2: chondroitin sulfate proteoglycan 2 (versican)
1388142_at	3.82	3.15	1.90	Cspg2: chondroitin sulfate proteoglycan 2 (versican)
1388265_x_at	1.75	2.60	2.28	Cspg2: chondroitin sulfate proteoglycan 2 (versican)
1388416_at	3.10	2.03	1.99	--
1388528_at	--1.50	--2.24	--1.43	Fbl: Fibrillarin
1389006_at	2.15	1.89	1.46	Mpeg1: member of the membrane attack complex
1389408_at	2.91	3.09	1.58	

Listed are probe sets for genes that showed significant difference between the arthritic and control rat blood identified by analysis of variance and filtered by empirical cutoffs. Probe set: identification of known genes and expressed sequence tags on the chip; Fold change: fold change values that was calculated between the arthritic samples and the time-matched controls; gene description: description of the genes encoded by the corresponding probe set.
